# Unravelling the Role of Electrochemically Active FePO_4_ Coating by Atomic Layer Deposition for Increased High‐Voltage Stability of LiNi_0.5_Mn_1.5_O_4_ Cathode Material

**DOI:** 10.1002/advs.201500022

**Published:** 2015-03-25

**Authors:** Biwei Xiao, Jian Liu, Qian Sun, Biqiong Wang, Mohammad Norouzi Banis, Dong Zhao, Zhiqiang Wang, Ruying Li, Xiaoyu Cui, Tsun‐Kong Sham, Xueliang Sun

**Affiliations:** ^1^ Department of Mechanical and Materials EngineeringUniversity of Western OntarioLondonONCanadaN6A 5B9; ^2^ Department of ChemistryUniversity of Western OntarioLondonONCanadaN6A 5B7; ^3^ Canadian Light SourceSaskatoonSKCanadaS7N 2V3

**Keywords:** X‐ray absorption spectroscopy, atomic layer deposition, iron phosphate, lithium‐ion batteries, lithium nickel manganese oxide

## Abstract

**Ultrathin amorphous FePO_4_ coating** derived by atomic layer deposition (ALD) is used to coat the 5 V LiNi_0.5_Mn_1.5_O_4_ cathode material powders, which dramatically increases the capacity retention of LiNi_0.5_Mn_1.5_O_4_. It is believed that the amorphous FePO_4_ layer could act as a lithium‐ions reservoir and electrochemically active buffer layer during the charge/discharge cycling, helping achieve high capacities in LiNi_0.5_Mn_1.5_O_4_, especially at high current densities.

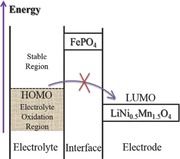

LiNi_0.5_Mn_1.5_O_4_ (LNMO) is a derivative of the commercialized spinel LiMn_2_O_4_ with Ni^2+^ and Mn^4+^ occupying two octahedral sites of 4a and 16d, respectively aimed at suppressing the intrinsic deficiencies such as the Jahn–Teller distortion of Mn^3+^ with the same theoretical capacity as LiMn_2_O_4_ (148 mAh g^–1^).^1^ In addition, since the working mechanism of LiNi_0.5_Mn_1.5_O_4_ is mainly the redox couple of Ni^2+^/Ni^4+^, the theoretical operating voltage reaches 4.7 V (vs Li^+^/Li) compared with 4.0 V for LiMn_2_O_4_ (vs Li^+^/Li).^1^, ^2^ Such a high voltage inevitably involves the aggressive oxidation of the electrolyte and the dissolution of transitional metals, which cause the capacity fading.^3^ In order to overcome these drawbacks, various strategies such as surface modifications using metal oxides and phosphates like Al_2_O_3_,^4^ ZnO,^5^ MgO,^6^ ZrO_2_,^7^ Li_3_PO_4_,^8^ and AlPO_4_ 9 have been studied. Most of these coatings are, however, still restricted to the poor conductivity and/or nonuniformity. The former deficiency results in poor kinetics during charging/discharging, while the latter does not provide full protection of electrode from HF attacking.^10^ Atomic layer deposition (ALD) is a novel coating technique capable of depositing highly conformal and uniform layers with well controlled thickness onto substrates.^11^ ALD‐derived ultrathin Al_2_O_3_ and LiAlO_2_ coatings have been used as protection layers on LNMO recently,^12^ it was found that the coating layer containing lithium favors faster lithium‐ion diffusion. Most of the nonlithium‐containing coating materials, however, increase the cycling stability at the expense of sacrificing capacity.^13^ For example, in their attempt to protect the surface of LNMO by ALD‐derived Al_2_O_3_, Jung et al.[[qv: 12b]] used only two ALD cycles of Al_2_O_3_ growth on LNMO powders, the capacity dropped by 10 mAh g^−1^ immediately, when the ALD cycle number was increased to 10, almost no capacity was delivered. In regard of this, the majority of previous studies deposited ALD layer onto the surface of electrode sheet instead of material powders so as to avoid the insulation between binder, conductive carbon, and cathode materials since the coating on electrodes did not break the contact between them.^14^ This will certainly restrict the application of ALD because some ALD materials require high deposition temperature, under which the binder may be unstable. Therefore, searching for a coating material with good electron and lithium diffusion, whilst protecting the cathode material uniformly under high voltage is exceptionally important. Despite the versatile design of ALD, coating materials that are electrochemically active, i.e., capable of accommodating Li^+^ within the voltage range of cathode materials are scarcely reported. With the electrochemically active advantage, direct coating onto powders becomes realistic.

Amorphous FePO_4_ has been widely studied as cathode materials for both lithium‐ion batteries (LIBs) and sodium‐ion batteries (SIBs).^15^ Its highly amorphous structure does not generate any lattice stress and therefore provides continuous lithium insertion channels and considerable electronic conductivity.^16^ In our recent work, we successfully synthesized FePO_4_ via ALD process,^17^, ^18^ which has also been demonstrated by Fjellvåg and co‐workers to deliver a discharge capacity of above 140 mAh g^–1^.^19^

In this study, we propose the novel ALD‐derived ultrathin amorphous FePO_4_ coating as a lithium‐ion reservoir during cycling, which may act as both a lithium diffusion facilitator and an electrochemical buffer layer between the electrolyte and LNMO by keeping the interface electrochemical potential above the electrolyte's highest occupied molecular orbital (HOMO), at which the electrolyte starts to get oxidized.^20^ Further, we provide detailed discussion about the role of electrochemically active FePO_4_ coating based on X‐ray absorption spectroscopy (XAS) analysis.

The preparation process of LNMO powders is described in the experimental section. The phases of LNMO were identified via XRD as shown in Figure S1 (Supporting Information). The peaks can be well indexed to the cubic spinel phase of LNMO (JCPDS No. 35–0782). ALD processes did not change the structures of the spinel LNMO. Due to the ultrathin and amorphous nature of the FePO_4_, no peaks of FePO_4_ can be observed in the XRD pattern. **Figure** **1**a shows the Raman spectra of the samples, the sharp peak at 160 cm^−1^ indicates that the LNMO is ordered P4_3_32 phase, with subtle oxygen deficiencies. The peaks at 400 and 490 cm^−1^ are related to the Ni^2+^–O stretching and the peak at 630 cm^−1^ corresponds to the Mn–O stretching of MnO_6_ octahedra.^21^ It can be seen that with increased ALD FePO_4_ cycles, both the Ni–O and Mn–O vibrations show blue shifts, this is due to the strains induced by the surface coating, which was also observed in TiO_2_ coating.^22^ In order to observe the P content evolution with ALD cycles, the P 2*p* spectra were collected by synchrotron XPS technique. It can be observed that these P atoms on the LNMO surface show increasing concentration when the ALD cycle increases, indicating that the amount of surface coating layer correlates to the number of ALD cycles. It is worthwhile to note that the P 2*p* XPS spectra of LNMO‐40 did not show linear intensity increase, this is probably due to the surface saturation in synchrotron XPS.

**Figure 1:**
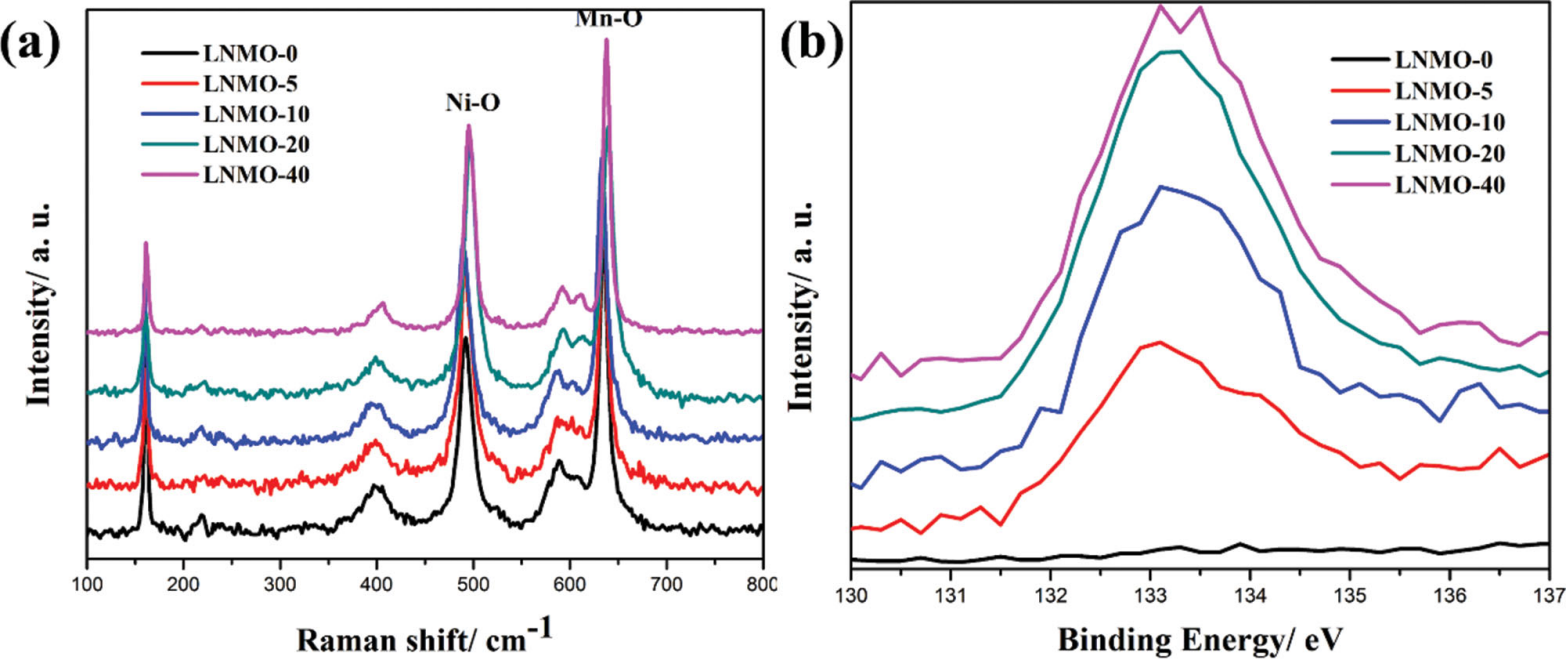
a) Raman spectra and b) P 2*p* XPS spectra of LNMO‐*n*.

The morphologies of the LNMO‐*n* samples were characterized by FESEM (**Figure** **2**a,b shows LNMO‐0 and LNMO‐20, the rest are shown in Figure S3, Supporting Information) and HRTEM (Figure 2c). It can be seen that the pristine LNMO shows sharp crystallized edges, the surface becomes rougher when the ALD cycle number increases, Figure S2 (Supporting Information) shows the EDX mapping of Fe, P, Mn, Ni, and O, it can be seen that the Fe and P are uniformly coated onto the surface of LNMO. The HRTEM images in Figure 2c reveal that the ultrathin surface coating is about 2 nm in thickness, the growth rate is consistent with our previous findings when depositing FePO_4_ onto Si wafer.^18^ The lattice fringe with basal distance of 0.24 nm is consistent with the (222) spacing of cubic phase LNMO. The inset electron diffraction pattern of LNMO‐20 indexes a typical spinel lattice structure.

**Figure 2:**
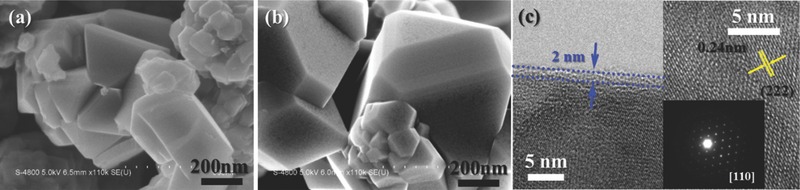
FESEM images of a) LNMO‐0 and b) LNMO‐20; c) HRTEM images of LNMO‐20 (inset: Electron diffraction patterns of the LNMO‐20 along the [110] zone axis).

**Figure** **3**a shows the first charge/discharge curves of LNMO‐*n* samples, the plateaus at around 4.7 V correspond to the reduction of Ni^4+^ to Ni^3+^ and Ni^2+^, another small plateau at around 4.0 V corresponds to the reduction of Mn^4+^ to Mn^3+^. The bare LNMO delivers highest first discharge capacity of 113 mAh g^−1^ among all the samples. Nevertheless, the LNMO‐0 sample decays rapidly during cycling, and the capacity retention of LNMO‐0 is only 79.89% after 100 cycles, as shown in Figure S5 (Supporting Information), Figure 3b and **Table** **1**. In contrast, the ALD FePO_4_‐coated samples display increasing capacity retention with more ALD cycle numbers, indicating the protective nature of the FePO_4_ layer.^23^ It is worthwhile to mention that despite the LNMO‐40 sample shows greatly enhanced stability, the capacity is lower, possibly due to the relatively lower electrical conductivity of FePO_4_. Rate capability test (Figure 3c) also reveals that LNMO‐10 presents the highest capacity under high current densities, e.g., more than 80 mAh g^−1^ at 5 C, while the LNMO‐0 sample drops to approximately 0 mAh g^−1^. The Coulombic efficiencies of the samples are shown in Figure S6 (Supporting Information), it can be seen that the Coulombic efficiency increases with the ALD cycle number, revealing that the presence of FePO_4_ has helped to suppress the electrolyte decomposition.

**Table 1:** Potentials of the oxidation/reduction peaks of the first CV scan, the capacity retentions and *R*_s_ after 100 charge/discharge cycles

LNMO‐*n*	Ni^2+^/Ni^3+^ [V]	Ni^3+^/Ni^2+^ [V]	Δ*V* [V]	Ni^3+^/Ni^4+^ [V]	Ni^4+^/Ni^3+^ [V]	Δ*V* [V]	Capacity retention [%]	*R*_s_ [Ω]
*n* = 0	4.777	4.637	0.140	4.825	4.682	0.143	79.89	173.1
*n* = 5	4.776	4.663	0.113	4.808	4.704	0.104	88.94	102.1
*n* = 10	4.760	4.651	0.109	4.805	4.693	0.112	91.96	91.7
*n* = 20	4.765	4.654	0.111	4.808	4.698	0.110	93.98	62.6
*n* = 40	4.750	4.668	0.082	4.794	4.710	0.084	100.00	57.9

**Figure 3:**
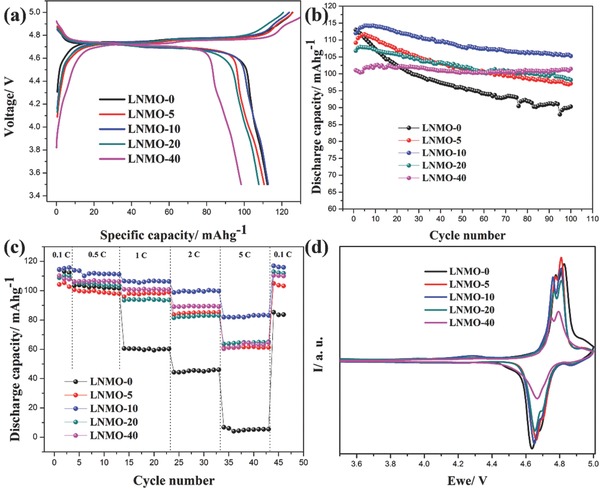
a) First charge/discharge curves; b) cycling stability under 0.5 C; c) rate capabilities; d) cyclic voltammetry of the LNMO‐*n* samples.

Cyclic voltammetry (CV) measurements were carried out on the LNMO‐*n* samples with normalized active material loading and electrolyte amount (Figure 3d). Three redox couples can be observed in the CV profiles. The weak and broad pair at around 4.0 V corresponds to the Mn^3+^/Mn^4+^, indicating that the LNMO is mostly in the phase of P4_3_32,^21^ in accordance with the Raman spectra. Two pairs of intense redox couples at 4.6–4.9 V are related to the Ni^2+^/Ni^3+^/Ni^4+^, which are the main sources of capacity. CV curves enlarged at 4.9–5.0 V (Figure S7, Supporting Information) show that the bare LNMO has much higher resident current value at the cutting voltage of 5.0 V than other samples, implying that the electrolyte oxidation in bare sample is more severe than coated samples. The lower area of the LNMO‐40 sample is also in accordance with the lower capacity. Table 1 summarizes the potential positions of the redox peaks. The redox peak potentials varied from 0.140 and 0.143 V for LNMO‐0 to 0.082 and 0.084 V for LNMO‐40 FePO_4_, suggesting that FePO_4_ coatings alleviates the polarization of the LNMO materials.

In the effort to understand the formation of solid electrolyte interface (SEI) on the surface of the electrodes, AC electrochemical impedance spectra (EIS) were conducted on each LNMO‐*n* sample after cycling for 100 times and subsequently charged to 5.0 V as shown in **Figure** **4**a. It can be seen that the LNMO‐0 sample shows only one semicircle, whereas those with FePO_4_ coatings show two semicircles in the range of high and medium frequencies. A possible equivalent circuit is proposed to illustrate the impedance behaviors on the surface as shown in Figure 4b. *R*_Ω_ stands for the Ohmic electrolyte resistance. The semicircle at high frequency is suggested to be a resistor *R*_s_ and a constant phase element (CPE), which are related to the migration of Li^+^ through the surface film, in this case, it reflects the resistance of SEI. Another semicircle at medium frequency is related to the charge transfer reaction composed of *R*_ct_ and another CPE, together with the finite length Warburg impedance.^24^ The values of the *R*_s_ are presented in Table 1, it can be found that without any FePO_4_ coating, the *R*_s_ for LNMO‐0 is 173.1 Ω, however, the existence of FePO_4_ coating layer helped to decrease the *R*_s_ values dramatically, which vary monotonically with the number of ALD cycles, to only 57.9 Ω for the LNMO‐40 sample. The drop of *R*_s_ clearly reveals the less formation of insulating SEI, which is a result of electrolyte decomposition, therefore FePO_4_ is effective towards suppressing the electrolyte decomposition.

**Figure 4:**
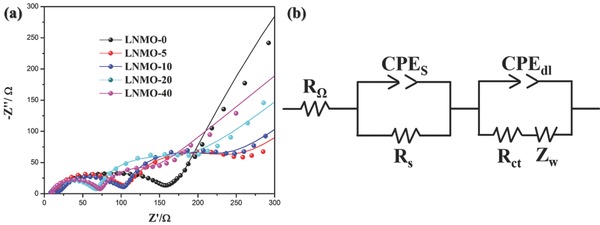
a) Electrochemical impedance spectra (EIS) of the LNMO‐*n* samples (Solid lines: Fitted spectra); b) a possible equivalent circuit.

To investigate the change of Mn valence state in the LNMO‐*n* samples, XANES was collected on the Mn L_3,2_‐edges. Mn L_3,2_‐edges illustrate the electronic transition from Mn 2*p*_3/2_ and 2*p*_1/2_ to an unoccupied 3*d* state.^25^ **Figure** **5**a depicts the total electron yields (TEY) of LNMO‐0 and LNMO‐20, which is surface sensitive with a probing depth of 5–10 nm. The L_2_‐edge often appears to be broader due to the core hole lifetime as explained by Coster–Kronig Auger decay.^26^ It can be seen that both the LNMO‐0 and the LNMO‐20 show predominantly Mn^4+^ features that fit well with standard MnO_2_, the small peak at 646 eV corresponds to Mn^3+^, and this is also consistent with the Raman spectra, the unchanged spectra reveal that the coating process did not generate changes to the surface phase of LNMO. However, after charge/discharge cycling, Mn^4+^ at the surface was partially reduced to Mn^2+^, and the LNMO‐0 shows much higher intensity ratio of Mn^2+^/Mn^4+^ than the coated LNMO sample. The bulk‐sensitive fluorescence yield (FYI) spectra of LNMO‐20, LNMO‐20 after battery cycling and LNMO‐0 after battery cycling are shown in Figure S8 (Supporting Information). It can be seen that the bulk Mn exhibits subtle changes after cycling. The less reduced Mn valence on coated LNMO surface also reveals weaker reduction by the electrolyte, which can be attributed to the inhibitive role of FePO_4_ against the electrolyte oxidation.^27^ It is also generally accepted that the presence of Mn^3+^ triggers the Jahn–Teller distortion because of its (*t_2g_* ^3^–*e_g_* ^1^) configuration, resulting in its charge disproportionation into non Jahn–Teller active Mn^2+^ and Mn^4+^, described as 2Mn^3+^ → Mn^2+^ + Mn^4+^.[[qv: 3a]],^28^ In the presence of HF from the LiPF_6_ salt, Mn^2+^ ions dissolve in the electrolyte and migrate through the separator followed by depositing on the anode as Mn metal, with a secondary phase formed on the surface of cathode materials.^29^ The suppression of Jahn–Teller distortion by FePO_4_ coating prevents the formation of Mn^2+^, thereby decreases the chance of Mn^2+^ dissolution in HF, and improves the stability of LNMO.^30^

**Figure 5:**
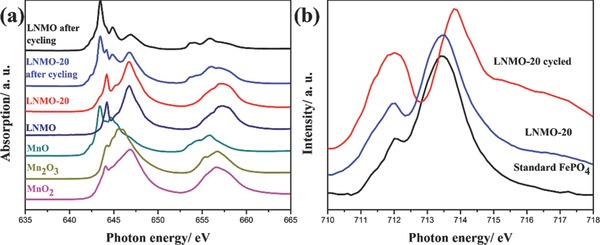
a) XANES Mn L_3,2_‐edges of LNMO‐20, LNMO‐20 cycled, LNMO‐0, LNMO‐0 cycled, and standard MnO, Mn_2_O_3_, MnO_2_; b) Fe L_3_‐edges of standard FePO_4_, LNMO‐20 and LNMO‐20 after 100 battery cycles collected in TEY mode.

Fe L_3_‐edges XANES of standard FePO_4_, LNMO‐20, and LNMO‐20 after battery cycling were performed to determine the chemical states of the FePO_4_ coatings before and after charge/discharge cycling. As shown in Figure 5b, the spectrum of LNMO‐20 fits well with the standard FePO_4_ spectrum, the intense peak at 713.5 eV (can be ascribed to the dominant spectral feature of Fe^3+^) and the weaker peak at 712.2 eV are related to the spin‐orbit, interplay of crystal‐filed, and electronic interactions. Their intensity ratio reveals the Fe^3+^/Fe^2+^ ratio.^31^ Nevertheless, upon battery cycling, there is an obvious drop in the Fe^3+^/Fe^2+^ ratio, indicating that part of the Fe^3+^ has been reduced, and the position of the right peak is, interestingly, shifting to higher energy value. Such shift was also observed in our previous study on the soft XANES spectroscopies of FePO_4_‐related various phases.^31^ In this regard, we believe that the insertion of lithium ions into the matrix of amorphous FePO_4_ has resulted in the partially lithiated FePO_4_ domains, which acts as a lithium‐ion reservoir and exhibited improved performance at high current densities by providing abundant Li^+^ diffusion pathways.

Based on the aforementioned results, the schematic illustration of the protecting role of FePO_4_ is presented in **Figure** **6**. The LNMO‐0 exposed to electrolyte suffers from side reactions such as fierce transitional metal dissolution and continuous electrolyte decomposition. On the contrary, LNMO with FePO_4_ coating is resistant to the side reactions. This is because it was found that the noncoated sample displayed Mn at reduced state on the surface after cycling, which is much more prone to dissolution compared with Mn^3+^ and/or Mn^4+^.[[qv: 29b]] Additionally, the amorphous FePO_4_ layer accommodates lithium ions rapidly during cycling, thus provides fast lithium diffusion. More specific role of FePO_4_ is shown in Figure 6b with the electrolyte's highest occupied molecular orbital (HOMO) and work functions of FePO_4_ and LiNi_0.5_Mn_1.5_O_4_. The electrolyte gets readily oxidized when the electrochemical potentials of cathode materials are below the HOMO of it.^20^ Unlike other conventional insulating ALD coating materials such as Al_2_O_3_ or ZrO_2_, and FePO_4_ is electrochemically active with an open‐circuit voltage of ≈3 V,[[qv: 15a]] the FePO_4_ ultrathin layer on the surface prevents the direct contact of LNMO with the electrolyte, helping to avoid the oxidation of electrolyte that results in the reduction and dissolution of Mn ions.

**Figure 6:**
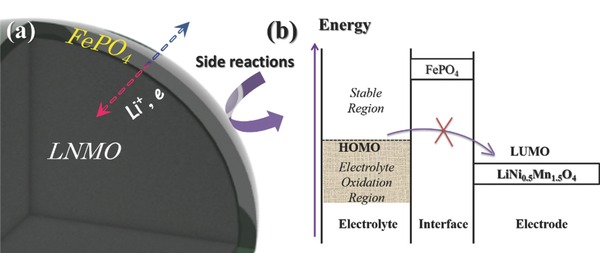
Schematic illustrations of a) LNMO‐*n* upon cycling; b) illustration of the electrolyte highest occupied molecular orbital (HOMO) and work functions of FePO_4_ and LiNi_0.5_Mn_1.5_O_4_.

We have proposed a new FePO_4_ coating on high voltage LNMO cathode material enabled by ALD. Different thicknesses of FePO_4_ have been deposited onto LNMO powders with 5, 10, 20, and 40 ALD cycles. The LNMO coated with 10 ALD cycles of FePO_4_ showed the best performance including the highest capacity and stabilized capacity retention under all the current rates. When the LNMO was coated with 40 ALD cycles of FePO_4_, the capacity retention increased up to 100%. XANES study showed that the ultrathin FePO_4_ suppressed the surface Mn^4+^ from being heavily reduced to Mn^2+^ by the reduction from the electrolyte and the Jahn–Teller distortion, less amount of Mn^2+^ helped to retain the surface consistency without severe dissolution into the electrolyte. The FePO_4_ coating layer was slightly reduced due to the remaining Li^+^ in the structure after charge/discharge cycling. Compared with the most widely used insulating Al_2_O_3_, amorphous FePO_4_ presents many advantages on the electron/ion diffusion on the surface. Our work provides an alternative option of depositing materials onto powders instead of electrode sheets directly using ALD, which expands the deposition temperature, owing to the electrochemically active nature of FePO_4_.

## Experimental Section

*Materials Synthesis*: LiNi_0.5_Mn_1.5_O_4_ was synthesized via a two‐step hydrothermal‐assisted carbonate precipitation method followed by thermal treatment. Ni(NO_3_)_2_·6H_2_O (99%, Aldrich, 0.005 mol) and Mn(NO_3_)_2_· 4H_2_O (99%, Aldrich, 0.015 mol) were dissolved in deionized water (5 mL). Na_2_CO_3_ (99%, Aldrich, 1 mol L^–1^, 20 mL) solution was subsequently added to the above mixture of nickel nitrate and manganese nitrate under vigorous stirring at a rate of 0.25 mL min^–1^, then the green precipitation was transferred to a 40 mL Teflon‐lined autoclave and kept at 140 °C for 10 h. After cooling down to room temperature (RT), the precipitation was filtered and washed with water several times and dried at 80 °C overnight. The carbonate powders were annealed at 450 °C for 4 h in air so as to obtain corresponding oxides. Thereafter, the oxide powders were mixed with Li_2_CO_3_ (99%, Sigma‐Aldrich, 0.00503 mol) in 1:1 water and ethanol mixture (10 mL) and left to dry under stirring at 60 °C. The mixed precursor was subsequently sintered in O_2_ at 800 °C for 6 h and then cooled to 600 °C in 3 h. After keeping at 600 °C for another 6 h, the furnace was cooled to RT at a cooling rate of 1 °C min^−1^ to obtain the final LNMO.

Amorphous FePO_4_ was deposited at 300 °C by using ferrocene (FeCp_2_, FeC_10_H_10_, 98% Sigma Aldrich), ozone (O_3_, 9.8 wt%), trimethyl phosphate (TMPO, (CH_3_O)_3_PO, 97% STREM Chemicals), and distilled water (H_2_O) as precursors in a Savannah 100 ALD system (Cambridge Nanotech, USA). The source temperature for FeCp_2_ and TMPO was 130 and 75 °C respectively. O_3_ and H_2_O were fed into the reactor chamber at RT. The deposition of FePO_4_ was achieved by following a sequence of FeCp_2_ pulse (1 s) – purge (10 s) – O_3_ pulse (1 s) – purge (10 s) – TMPO pulse (2 s) – purge (10 s) – purge (10 s) – H_2_O pulse (1 s) – purge (10 s). Nitrogen gas (99.999%) was used as a carrying and purging gas at a flow rate of 20 sccm. The above processes were repeated for several (*n*) times to grow *n* cycles of FePO_4_ onto LNMO powders, denoted as LNMO‐*n* (bare LNMO when *n* = 0).

*Characterization Methods*: The morphology of LNMO‐*n* was characterized by a Hitachi S‐4800 field emission scanning electronic microscopy (FESEM) equipped with an energy dispersive X‐ray spectroscope (EDS), Hitachi H‐7000 transmission electron microscope (TEM), and a high‐resolution transmission electron microscope (HRTEM, JEOL 2010F). Raman scattering (RS) spectra was collected from a HORIBA Scientific LabRAM HR Raman spectrometer system with a 532.4 nm laser and optical microscope at RT. X‐ray diffraction (XRD) patterns were collected on a Bruker D8 Advance Diffractometer using Cu K_α_ radiation at 40 kV and 40 mA. The X‐ray absorption near edge structure (XANES) measurements at total electron yield (TEY) and fluorescence yield (FYI) modes of Mn L_2,3_‐edge and Fe L_3_‐edge were performed at the Canadian Light Source (CLS) on the high resolution Spherical Grating Monochrometer (SGM) beamline using a 45 mm planar undulator and three gratings with a photon energy range of 250–2000 eV, LNMO‐20 was chosen as the target sample. The P 2*p* X‐ray photoemission spectroscopy (XPS) was performed at the variable line spacing plane grating monochromator (VLS PGM) beamline at 200 eV photon energy with a total resolution of 100 meV.

*Electrochemical Measurements*: The LNMO‐*n* powders were mixed with poly(vinylidene fluoride) binder and acetylene black in a ratio of 8:1:1 in N‐methyl‐pyrrolidione (NMP) solvent to form slurries. The slurries were subsequently casted onto aluminum foils as the current collector and dried at 80 °C under vacuum overnight. The electrode was assembled in an Ar‐filled glovebox with moisture and oxygen concentrations below 1 ppm. A CR‐2032 type coin cell using a lithium metal as the counter electrode and Celgard K2045 as the separator was utilized. The electrolyte was composed of 1 m LiPF_6_ salt dissolved in ethylene carbonate (EC) and dimethyl carbonate (DMC) in a 1:1 volume ratio (BASF Corp.). Cyclic voltammetry (CV) was performed on a versatile multichannel potentiostat 3/Z (VMP3), with a scanning rate of 0.1 mV s^−1^ and a potential range of 3.5−5.0 V (vs Li^+^/Li) at RT. Electrochemical impedance spectroscopy (EIS) was also performed on the versatile multichannel potentiostat 3/Z (VMP3) by holding the cells at 5.0 V. Galvanostatical charge/discharge was performed on Arbin BT2000 at various current densities between 3.5 and 5.0 V (vs Li^+^/Li), the stability performance test was done under 0.5 C, which is 73.5 mA g^–1^.

## Supporting information

As a service to our authors and readers, this journal provides supporting information supplied by the authors. Such materials are peer reviewed and may be re‐organized for online delivery, but are not copy‐edited or typeset. Technical support issues arising from supporting information (other than missing files) should be addressed to the authors.

Supplementary
