# Supplementary material for: Unravelling the Role of Electrochemically Active FePO4 Coating by Atomic Layer Deposition for Increased High‐Voltage Stability of LiNi0.5Mn1.5O4 Cathode Material
Source: Adv Sci (Weinh). 2015 Mar 25;2(5):1500022. doi: 10.1002/advs.201500022 (PMC5115369; doi:10.1002/advs.201500022)
Supplement: Supplementary file 1 — Supplementary [file ADVS-2-0p-s001.pdf]

Copyright WILEY-VCH Verlag GmbH & Co. KGaA, 69469 Weinheim, Germany, 2015.

ADVANCED SCIENCE
Open Access

Supporting Information

for *Adv. Sci.*, DOI: 10.1002/advs.201500025

Unravelling the Role of Electrochemically Active $FePO_4$ Coating by Atomic Layer Deposition for Increased High-Voltage Stability of $LiNi_{0.5}Mn_{1.5}O_4$ Cathode Material

*Biwei Xiao, Jian Liu, Qian Sun, Biqiong Wang, Mohammad Norouzi Banis, Dong Zhao, Zhiqiang Wang, Ruying Li, Xiaoyu Cui, Tsun-Kong Sham, and Xueliang Sun**

Unravelling the role of electrochemically active $FePO_4$ coating by atomic layer deposition for increased high-voltage stability in $LiNi_{0.5}Mn_{1.5}O_4$ cathode material

Biwei Xiao,[a] Jian Liu,[a] Qian Sun,[a] Biqiong Wang,[a] Mohammad Norouzi Banis,[a] Dong Zhao,[b] Zhiqiang Wang,[b] Ruying Li,[a] Xiaoyu Cui,[c] T.-K Sham[b] and Xueliang Sun*[a]

[a]*Department of Mechanical and Materials Engineering, University of Western Ontario, London, ON, Canada N6A 5B9. E-mail: xsun@eng.uwo.ca; Tel: +1 5196612111 ext. 87759*

[b]*Department of Chemistry, University of Western Ontario, London, ON, Canada, N6A 5B7*

[c]*Canadian Light Source, Saskatoon, SK, Canada, S7N 2V3*

Supplementary Information

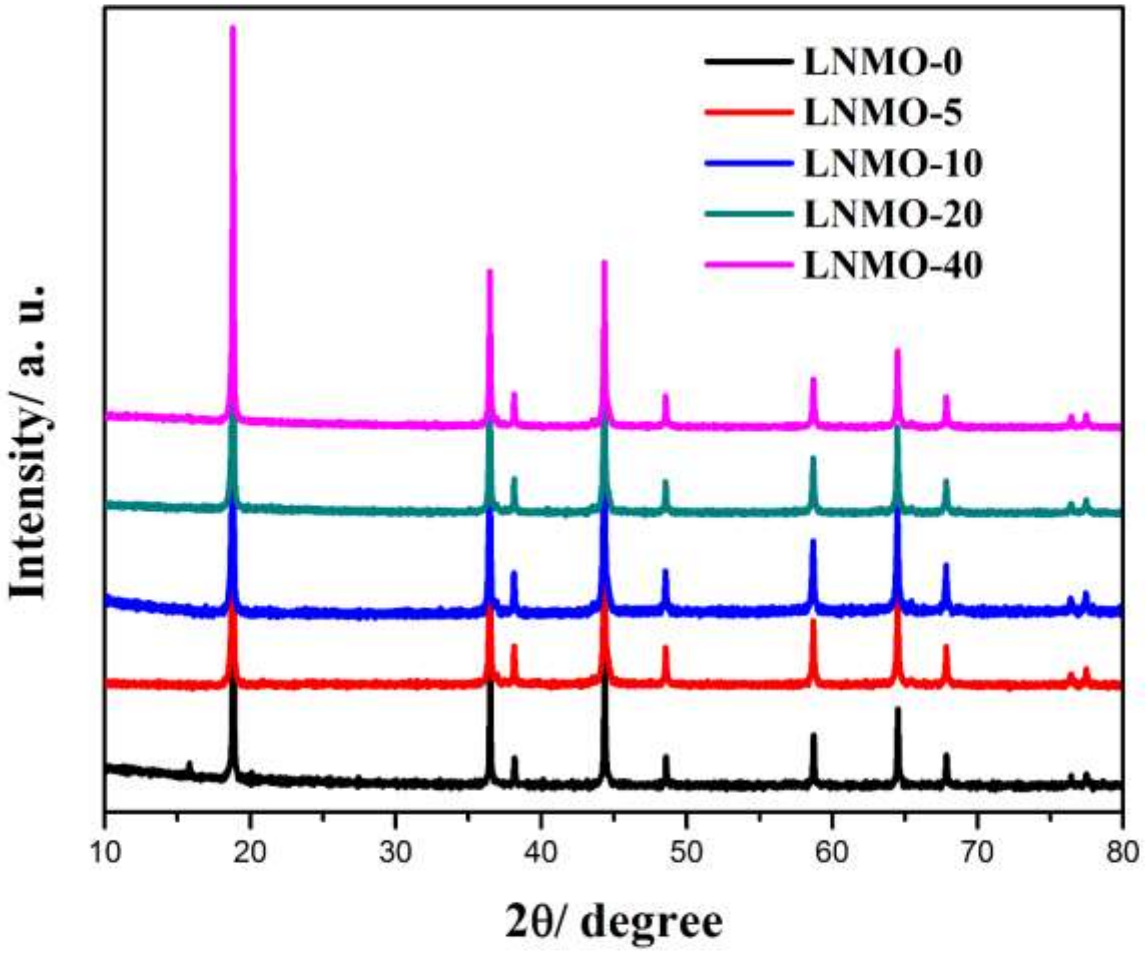

Fig. S1 X-ray diffraction patterns of LNMO/ n $FePO_4$

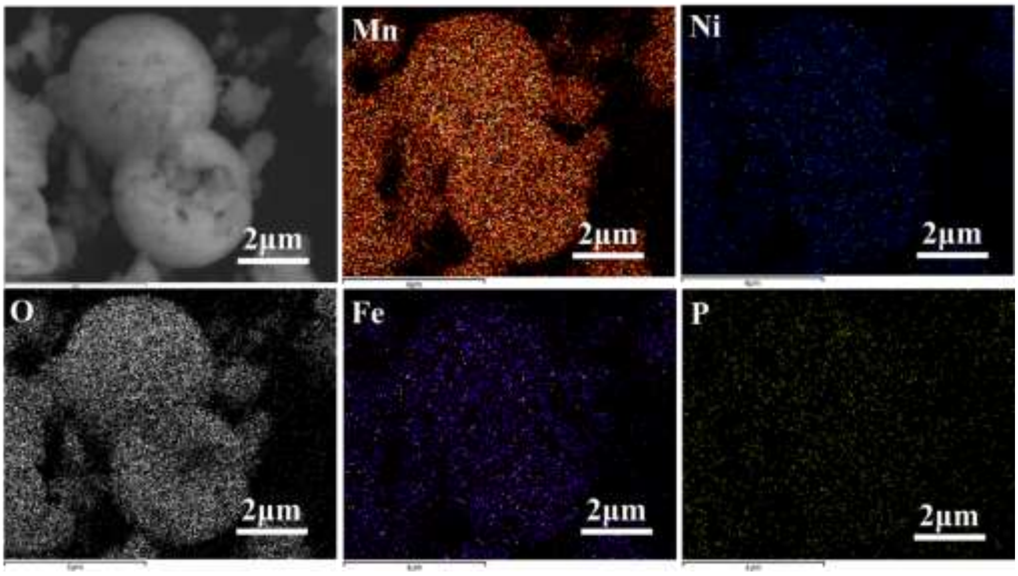

Fig. S2 Energy-dispersive x-ray (EDX) mapping of LNMO-20

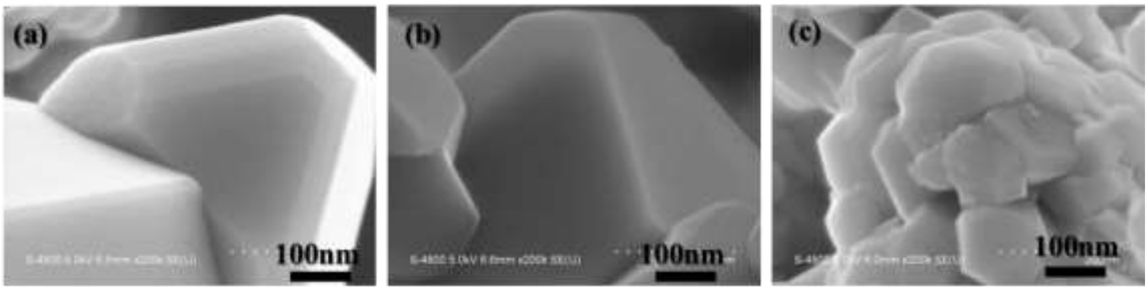

Fig. S3 SEM images of (a) LNMO/5 $FePO_4$; (b) LNMO/10 $FePO_4$; (c) LNMO/40 $FePO_4$

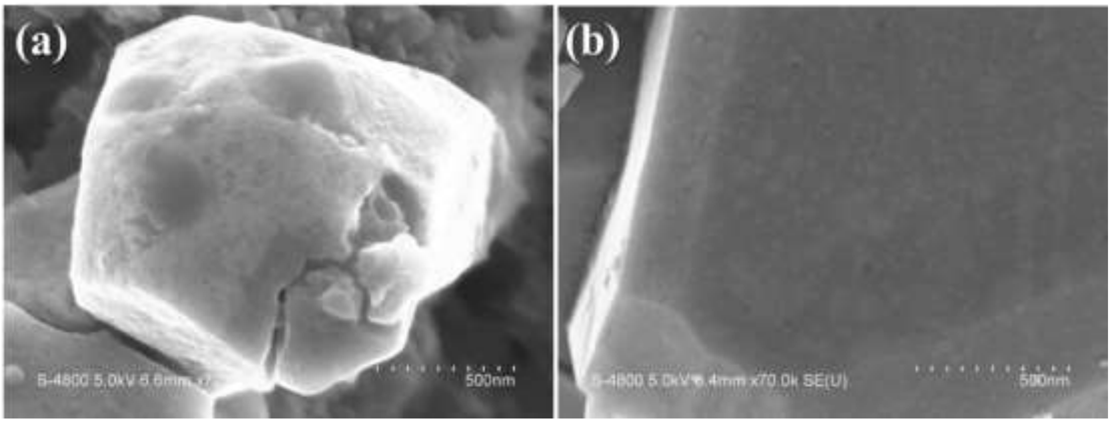

Fig. S4 SEM images of (a) bare LNMO and (b) LNMO-20 after 100 times battery cycling

The surface of bare LNMO is obviously covered by another layer of SEI, also, cracking can be observed due to the lattice volume expansion. Instead, the LNMO-20 $FePO_4$ sample does not show other depositions, indicating the less SEI formation.

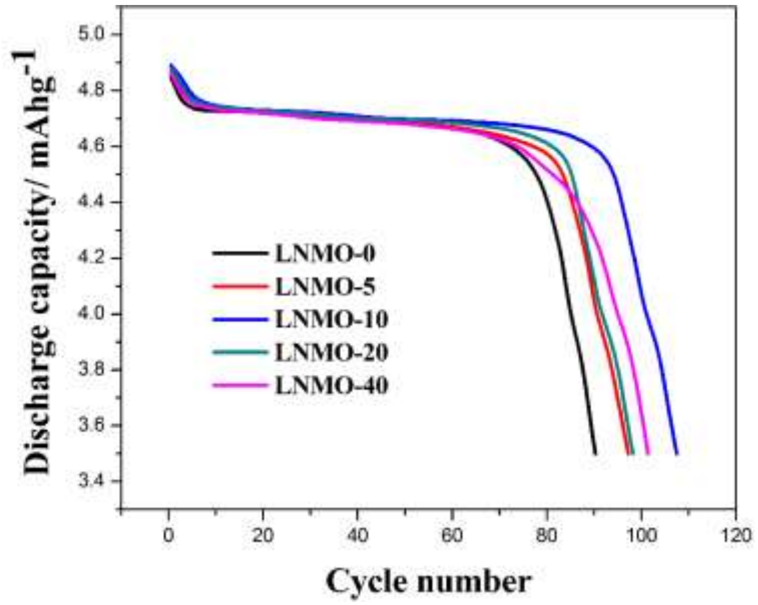

Fig. S5 Discharge curves of the 100th electrochemical cycle of the LNMO-n samples

The 100th discharge curves of the samples show the same trend with the stability test, in which LNMO-10 shows the highest capacity, the voltage plateau is also higher than the others, indicating its superior performance.

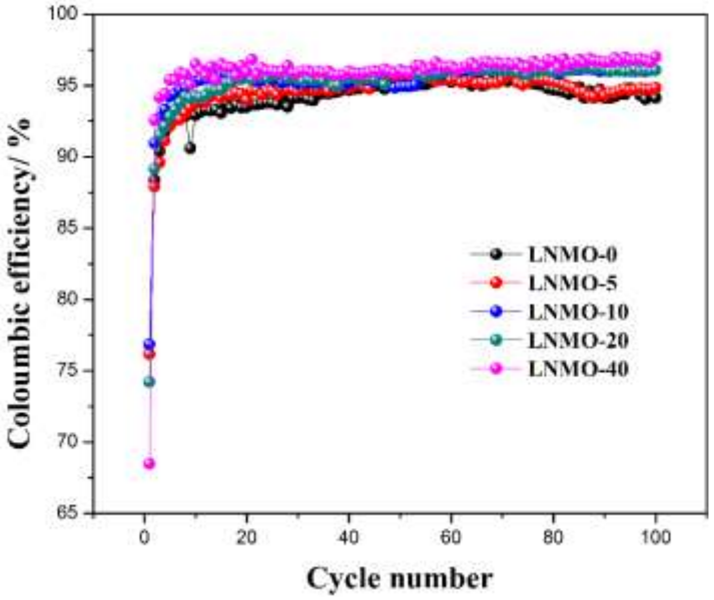

Fig. S6 Coulombic efficiency of the samples

It can be observed that the initial Coulombic efficiency (ICE) decreases with the increase of ALD cycles, this is because some of the lithium were trapped in the matrix of $FePO_4$ during the first charge from an open circuit voltage (OCV) of about 3.0 V. They were not reversible because the discharge cutoff voltage was 3.5 V, which is at the end of the $FePO_4$ electrochemically active window. This loss of lithium was responsible for the decreased ICE.

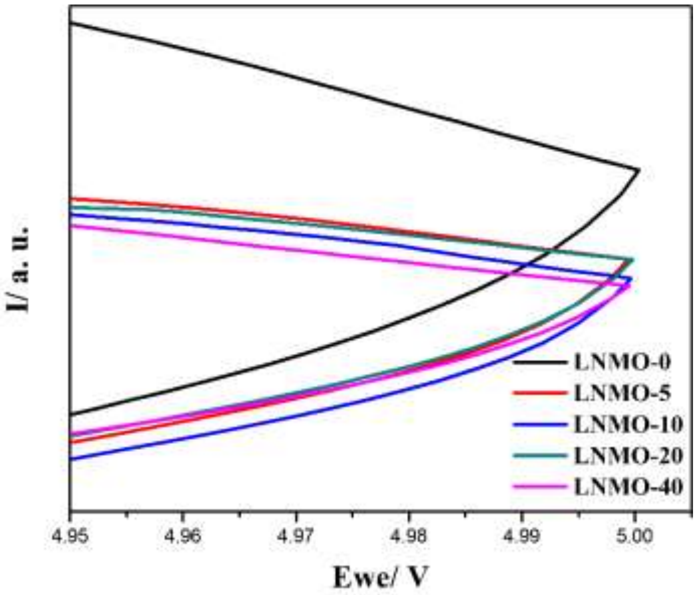

Fig. S7 Enlarged CV curves within 4.9 to 5.0 V

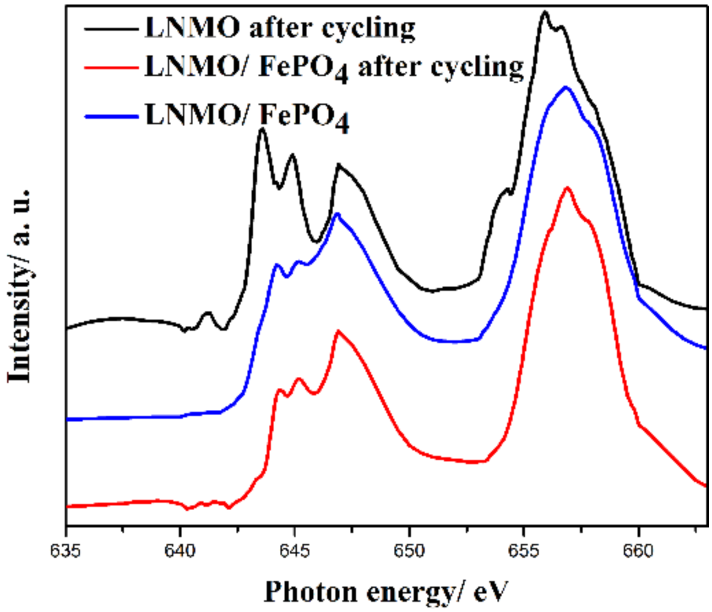

Fig. S8 Mn $L_{3,2}$-edge fluorescence yield (FYI) spectra of LNMO-20, LNMO-20 after battery cycling and bare LNMO after battery cycling

FYI is a bulk-sensitive technique, Fig. S8 indicates that the bulk Mn will be slightly reduced to lower valence without coating. Similar with the surface Mn, coating helps to retain Mn at higher oxidation level in the bulk, thus preventing the vigorous Mn^{3+} Jahn-Teller distortion and Mn^{2+} dissolution. The FYI reveals that the reduction of Mn in LNMO mainly takes place at the outer surface, where the cathode material is exposed to either the coated $FePO_4$ or the liquid electrolyte.
